# Characterization of the dsDNA prophage sequences in the genome of *Neisseria gonorrhoeae *and visualization of productive bacteriophage

**DOI:** 10.1186/1471-2180-7-66

**Published:** 2007-07-05

**Authors:** Andrzej Piekarowicz, Aneta Kłyż, Michał Majchrzak, Monika Adamczyk-Popławska, Timothy K Maugel, Daniel C Stein

**Affiliations:** 1 Institute of Microbiology, Warsaw University, Miecznikowa 1, 02-096 Warsaw, Poland; 2 Department of Cell Biology and Molecular Biology, University of Maryland, College Park, MD, 20742, USA; 3 Laboratory of Biological Ultrastructure, University of Maryland, College Park, MD, 20742, USA

## Abstract

**Background:**

Bioinformatic analysis of the genome sequence of *Neisseria gonorrhoeae *revealed the presence of nine probable prophage islands. The distribution, conservation and function of many of these sequences, and their ability to produce bacteriophage particles are unknown.

**Results:**

Our analysis of the genomic sequence of FA1090 identified five genomic regions (NgoΦ1 – 5) that are related to dsDNA lysogenic phage. The genetic content of the dsDNA prophage sequences were examined in detail and found to contain blocks of genes encoding for proteins homologous to proteins responsible for phage DNA replication, structural proteins and proteins responsible for phage assembly. The DNA sequences from NgoΦ1, NgoΦ2 and NgoΦ3 contain some significant regions of identity. A unique region of NgoΦ2 showed very high similarity with the *Pseudomonas aeruginosa *generalized transducing phage F116. Comparative analysis at the nucleotide and protein levels suggests that the sequences of NgoΦ1 and NgoΦ2 encode functionally active phages, while NgoΦ3, NgoΦ4 and NgoΦ5 encode incomplete genomes. Expression of the NgoΦ1 and NgoΦ2 repressors in *Escherichia coli *inhibit the growth of *E. coli *and the propagation of phage λ. The NgoΦ2 repressor was able to inhibit transcription of *N. gonorrhoeae *genes and *Haemophilus influenzae *HP1 phage promoters. The holin gene of NgoΦ1 (identical to that encoded by NgoΦ2), when expressed in *E. coli*, could serve as substitute for the phage λ *s *gene. We were able to detect the presence of the DNA derived from NgoΦ1 in the cultures of *N. gonorrhoeae*. Electron microscopy analysis of culture supernatants revealed the presence of multiple forms of bacteriophage particles.

**Conclusion:**

These data suggest that the genes similar to dsDNA lysogenic phage present in the gonococcus are generally conserved in this pathogen and that they are able to regulate the expression of other neisserial genes. Since phage particles were only present in culture supernatants after induction with mitomycin C, it indicates that the gonococcus also regulates the expression of bacteriophage genes.

## Background

The sequencing of bacterial genomes has revealed the presence of integrated viral genomes (prophages) in most of the sequenced bacterial genomes [1-3] Prophage DNA sequences can constitute up to 10–20% of the bacterial genome and are major contributors for differences between individual species [3]. Prophage gene expression may influence the pathogenicity or the general fitness of the bacterium [4,5]. The list of genes regulated by bacteriophage is very long and represents a broad group of genes (For review, see Brüsow *et al*. [1,6]).

Analysis of prophage DNA suggests that integration into bacterial genomes can lead to changes including inactivating point mutations, genome rearrangements, modular exchanges, invasion by further mobile DNA elements, and massive DNA deletion [2,6,7]. Bacteriophage have been described that can produce plaques on certain commensal Neisseria [8-10]. Bacteriophage able to propagate in *N. meningitidis *have been identified, but they were not able to propagate on other *Neisseria *strains [11]. Similarly, the presence of autoplaquing in *N. gonorrhoeae *was observed but no phage propagation was seen [12].

DNA sequence analysis has identified prophage DNA sequences in the genomes of most bacteria. In *N. meningitidis*, they belong to two groups of phages. The presence of the Mu-like prophage sequences was detected in the genomes of serogroup A strains of the epidemic subgroups I, III, IV-1 and VI of *N. meningitidis *[13-15]. Two additional Mu-like sequences were found in *N. meningitidis *serotype A [14]. A ~39.3-kb region named as Pnm1 [13] looks as if it could also encode for a functional bacteriophage. A sequence homologous to Pnm1 was found in the genome of serogroup B *N. meningitidis *but not in genome of *N. gonorrhoeae *FA1090 [15]. The second group consists of filamentous prophage sequences homologous to f1 and CTXΦ [16]. These prophages can excise, albeit with very low frequency, from the bacterial genome resulting in the production of biologically active phages [16].

In this paper we describe properties of prophage sequences present in *N. gonorrhoeae *genomes (NgoΦ1 – 5) that all belong to dsDNA tailed group of bacteriophage. We show the biological activity of some of the prophage genes and the presence of the prophage DNA sequences in bacterial cultures. We further demonstrate the production of phage particles by gonococci. However, we were unable to demonstrate the production of plaques on any of the *N. gonorrhoeae *or on non pathogenic *Neisseria *strains tested.

## Results

### Overall genetic structure of NgoΦ1 – NgoΦ5 prophages

**Figure 1:**
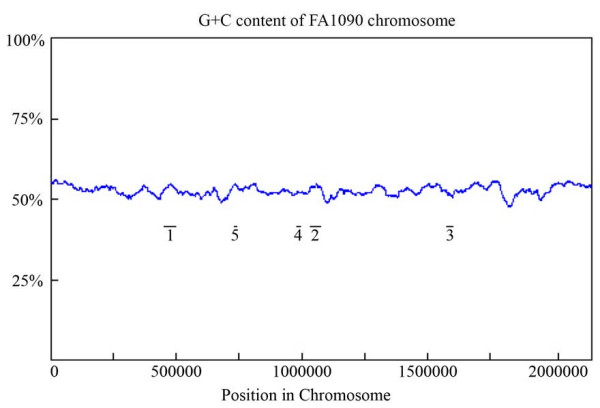
**G + C content of gonococcal chromosome**. The position of the various prophage sequences in the gonococcal chromosome are shown as short black lines, labeled 1 through 5. These bars represent prophages NgoI-5 respectively.

Annotation of the coding sequences in the published genomic sequence of *N. gonorrhoeae *strain FA1090 [17] indicated that the gonococcus possesses 5 genomic regions that encode proteins with significant homology to proteins found in dsDNA bacteriophages. The location of the coding sequences of each of these genomic regions is described in table 2. We have designated these regions NgoΦ1 to NgoΦ5. We determined the overall distribution of guanine and cytosine residues in the gonococcal chromosome, determined the G+C content by the windows sliding method for the putative prophage sequences, and identified their location on the gonococcal chromosome (See Fig. 1). Four of the prophage sequences have a higher G + C content (54–57%) while one (NgoΦ4) has a lower G + C content (49%) as compare to an average G + C content of 52.5% of *N. gonorrhoeae *genomic DNA. The location of each of the prophage coding sequences, while differing in G+C content from the overall gonococcal chromosome, did not appear to be significantly different from the regions immediately adjacent to the prophage sequences. We analyzed the codon usage for the predicted ORFs of the prophage regions and compared them to the overall codon usage of the gonococcus. While some of the ORFs appeared to have significant codon usage differences, the variations were no greater than what was obtained when we generated a codon usage profile for the two beta subunits of RNA polymerase (data not shown). This observation prevented us from drawing any evolutionary inferences, based on base content or codon usage.

**Table 2:** Localization of the prophage islands on the genome sequence of *N. gonorrhoeae *FA1090

**Prophage**	**Sequence coordinates**^a^	**Length of the DNA sequence (bp)**	**CDS annotations (Acc. No **AE004969)	**Number of CDS**^#^
ds DNA prophage sequences				

NgoΦ1	455173 – 498100	42 927	NGO0462-NGO0524	63
NgoΦ2	1044447 – 1078281	33 834	NGO1085-NGO1132	48
NgoΦ3	1583028 – 1599049 1606828 – 1609808	16 021 2 980	NGO1613-NGO1640 NGO1649-NGO1652	284
NgoΦ4	972837 – 984008	11 171	NGO1000-NGO1020	21
NgoΦ5	721256 – 729870	8 614	NGO0720-NGO0732	13

**Filamentous ss DNA prophage sequences**				

NgoΦ6	1080185 – 1088420	8 235	NGO1137 – NGO1146	13
NgoΦ7	1215967 – 1223383	7 416	NGO1262 – NGO1270	12
NgoΦ8	1103017 – 1109444	6 427	NGO1164 – NGO1170	8
NgoΦ9	1599378 – 1607537*	7 159	NGO1641 – NGO1648	9

^a^Coordinates are relative to the DNA sequence contained at **GenBank Acc. No **AE004969.

^# ^Number ofCDSdetermined in this paper for NgoΦ6, NgoΦ7, NgoΦ8 and NgoΦ9 is higher than cited by National Center for Biotechnology Information.

Note that the sequence of NgoΦ9 is inserted within the sequence of NgoΦ3

**Figure 2:**
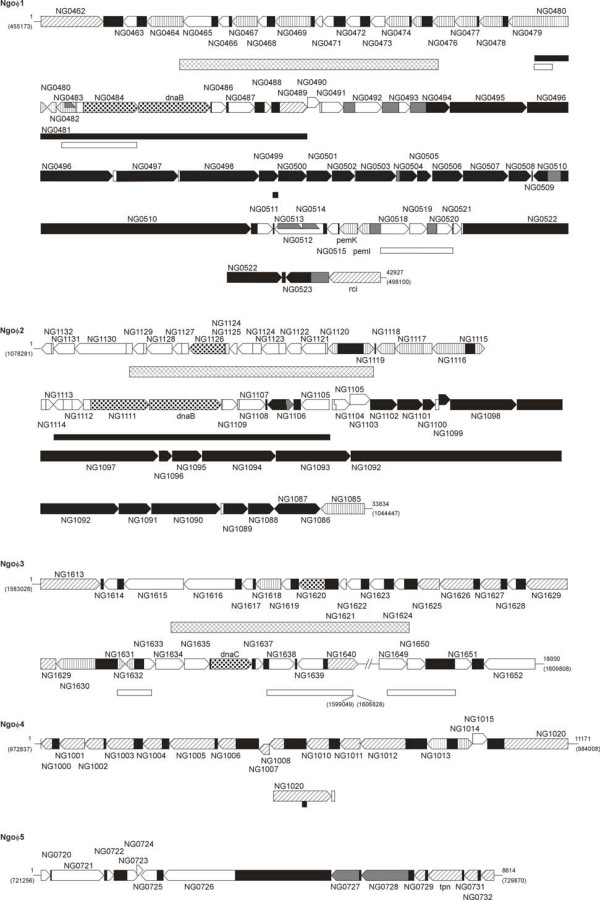
**Genomic organization of Ngoφ1 – Ngoφ5 prophages in *N. gonorrhoeae*. Arrows oriented in the direction of transcription represent CDSs or genes**. Arrows with black dots represents the replication module, solid arrows represent the structural module, and the arrows with vertical lines represent the CDSs encoding the repressors and other regulatory genes. The open arrows represent the CDSs with unknown function. Arrows with cross-hatching represent integrases. The filled rectangles (black or gray) represent putative non coding regions. The regions homologous for NgoΦ1, NgoΦ2 and NgoΦ3 are shown as rectangles with cross-hatched regions. The regions homologous for NgoΦ1 and NgoΦ2 are shown as solid black rectangles while those homologous for NgoΦ1 and NgoΦ3 as white open rectangles. The broken line representing the genome of NgoΦ3 (between CDSs 1640 and 1649) shows the site where the integrity of this genome is broken by the presence of part of the NgoΦ7 genome). The numbers at the beginning and end of each phage show the lengths of prophage genomes (in bp) while the numbers in parenthesis show the positions of prophages in *N. gonorrhoeae *FA1090 chromosome.

The five prophage sequences homologous with dsDNA phages ranged from 8614 bp to 42927 bp. BLAST and MegaBlast (nucleic acid) searches performed against the non-redundant database did not shown homology to any DNA sequences, except to very small regions in the genomes of *N. meningitidis *and *Pseudomonas aeruginosa *phage F116. This analysis also showed that the two, almost identical (with about 95% of identity) blocks of DNA, (of total length about 11000 bp) present at the left hand end of NgoΦ1 are present in the sequence of NgoΦ2 and one of them is also present in the sequence of NgoΦ3 (Fig. 2). These blocks include genes whose predicted function would be either regulatory or structural. The genome of NgoΦ3 is divided into two parts due to the presence of the inserted genomic sequence of a filamentous phage (Nf4-NgoΦ6). The NgoΦ3 prophage sequence was probably derived from NgoΦ1 or NgoΦ2. The sequences of NgoΦ4 and NgoΦ5 are not homologous and do not share any homology with any of the other phages.

We analyzed the protein coding sequences (CDS) for all five putative prophage sequences. Putative ORFs greater than 40 amino acids were identified using a combination of CDS identification programs. The results of this analysis show that the identified prophage islands contain CDSs with striking amino acid similarities to known functional phage proteins (Table 3). The length and the coding capacity of genomic DNA of NgoΦ1 and NgoΦ2 suggest that they could represent biologically active prophages. We also noted the presence of four filamentous ssDNA-like prophage sequences that share very high homology to such sequences in the *N. meningitidis *genome (homology ranged from 90–97% identity, data not presented). Since the genomic organization of these phage have been described for a variety of neisserial stains [16,18], we did not focus our studies on them.

**Table 3:** Properties of the NgoΦ1 CDS' sequences

NgoΦ1 CDSs	Genome position	Length (aa)	Putative function	Significant matches to proteins in GenBank, scores represent filtered BLASTP, % identities, over stated number of amino acid)	Identity with CDSs	
					
					NgoΦ2	NgoΦ3
NGO0462	455173–456369	398	phage integrase	VC0847 [*Vibrio cholerae*] 31, 51% on 406, Int A *E. coli *K12 32, 51% on 412		
NGO0463	456725–456994	89	NS*			
NGO0464	457189–457872	227	hypothetical protein	B3 CDS5 [Bacteriophage B3] 29, 46% on 227	1130, 1131	2e+07
NGO0465	457883–458419	178	NS			
NGO0466	458530–458745	71	NS		1129	1617
NGO0467	458797–459288	163	hypothetical protein	CDS8 [*Streptococcus *phage Φ-O1205] (pfam SiphoGp 35, 56% on 144, CDS157 [*Streptococcus *157 family) *Thermophilus *phage Sfi21] 31, 51% on 160	1128	1618
NGO0468	459285–459467	60	NS		1127	1619
NGO0469	459607–460293	228	replication initiation	BBTVs1gp1 [Banana bunchy top virus] replication protein 26, 43% on 109 primase A2p38 [*Lactobacillus casei *phage A2] 32, 44% on 87	1126	1620
NGO0470	460362–460523	53	NS		1125	1621
NGO0471	460520–460735	71	hypothetical protein	NMB0904 [*N. meningitidis *MC58] 45, 63% on 61, gp30 [*Burkholderia cepacia *phage Bcep22] 37, 54% on 48	1124	1622
NGO0472	460949–461236	95	NS		1123	1623
NGO0473	461422–461697	91	NS		1122	
NGO0474	461694–462170	158	transcriptional regulator	IrgB [*V. cholerae *strain N16961 serogroup O1] 26, 62% on 50	1121	
NGO0475	462203–462403	66	NS		1120	1624
NGO0476	462601–463014	137	NS			
NGO0477	463011–463448	145	transcriptional	EF1886 [*Enterococcus faecalis *(pfam HTH 3 family) regulator V583] 45, 70% on 44, NMB1204 [*N. meningitidis *MC58] 32, 59% on 62		
NGO0478	463489–463926	145	NS			
NGO0479	464039–464755	238	repressor protein cI	cI [*Salmonella typhimurium *(pfam peptidase 1630 phage T104] 29, 52% on 222 S24family), HK022 p43 [*Enterobacteria *phage HK022] 30, 49% on 223 [*Pseudomonas *phage D3] 26, 42% on 221		1630
NGO0480	465140–465295	51	NS		1114	1631
NGO0481	465272–465460	62	NS		1113	1632
NGO0482	465465–465827	120	NS			
NGO0483	46633–465860	75	hypothetical protein	Bcep22p22 [*B. cepacia *phage Bcep22] 32, 52% on 71	1112	1633
NGO0484	465978–467042	354	DNA replication protein	[Phage Aaphi23]33, 45% on 342 Gp54 [Phage HK97] 26, 42% on 123 [*Enterobacteria *phage P22] 24, 17% on 125	1111	
NGO0485	467039–468400	453	replicative DNA helicase	(pfam DnaB family, DnaB-C family) [*Enterobacteri*a phage P1] 34, 53% on 446, CDS74 [*Pseudomona*s phage D3 ] 32, 55% on 430	1110	
NGO0486	468417–468701	94	NS		1109	
NGO0487	468740–469234	164	hypothetical protein	SA1787 [*Staphylococcus *phage phiN315] 41, 51% on 58	1108	1638
NGO0488	469411–469560	49	NS		1106	
NGO0489	469696–470241	181	Endodeoxyribo-nuclease	rus [Phage 82] 34, 47% on102 (pfamRusA family) RUS Sb45 [*S. typhimurium *phage ST64B ] 31, 44% on 113, rus [Phage HK620] 30, 47% on 96		1640
NGO0490	470231–470464	77	hypothetical protein	gp18 [*B. cepacia *phage Bcep22] 46, 58% on 41		
NGO0491	470508–470915	135	NS			
NGO0492	471138–471644	168	NS			
NGO0493	471965–472186	73	hypothetical protein	SfVp44 [*Shigella flexner*i phage V] 50, 67% on 34		
NGO0494	472479–472928	149	terminase small subunit	f6p01 [*Enterobacteria *phage Sf6] 45, 69% on 122, 3 [Phage HK620] 45, 69% on 122		
NGO0495	472990–474411	473	terminase large subunit	T1p5324 [*Enterobacteria *phage T1] (COG 24, 42% on 505, TerL [Phage terminase Aaphi23] 28, 45% on 438,		
NGO0496	474408–476555	715	phage portal protein	gp34 [Phage phi-C31] (pfam Phage Mu F family) 25, 43% on 254, gp34 [Phage phiB 25], 43% on 258		
NGO0497	476623–477780	385	protease structural protein	T5 150 [Phage T5] 31, 48% on 137 psiM100p19 [*Methanothermobacter wolfeii *prophage psiM100], 46% on 140		
NGO0498	477821–479320	499	hypothetical protein	2 [*Equine herpesvirus *1] 23, 30% on 18		
NGO0499	479327–479689	120	NS			
NGO0500	479692–480222	176	hypothetical protein	gp8 [Phage phiE125] 30, 44% on 125		
NGO0501	480370–480702	110	putative virion	SO690 protein, prophage MuSo2, morphogenesis protein [*Shewanella oneidensis *MR-1] 47, 60% on 53		
NGO0502	480699–481127	142	NS			
NGO0503	481153–481926	257	NS			
NGO0504	481987–482316	109	hypothetical protein	PHG31p11 protein [*Aeromonas *phage 31] 28, 46% on 88		
NGO0505	482328–482594	88	NS			
NGO0506	482594–483193	199	hypothetical protein	gp83 [Phage phi JL001] 25, 46% on 80		
NGO0507	483190–484044	284	hypothetical protein	gp84 [Phage phi JL001] 42, 58% on 103, D3112p50 [Phage D3112] 32, 47% on 50		
NGO0508	484046–484477	143	hypothetical protein	BMEI1342 protein) (pfam06940, DUF1287 [*Brucella melitenis*] 33, 47% on 119		
NGO0509	484505–484789	93	unknown	CDS114 [*Pseudomonas *phage phiKZ 43, 60%on 81 transcriptional regulator PP0276 [*P. putida *KT2440] Cro/cI family 39, 65% on 63		
NGO0510	485026–489171	1381	hypothetical protein	gp86 [Phage phi JL001] 22,39% on 459 CDS97 [*Lactobacillus plantarum *phage LP65] 26, 42% on 173 putative phage tail protein HCM2.005c protein [*S. enterica*] 27, 44% on 180		
NGO0511	489283–489588	101	NS			
NGO0512	489593–490528	311	NS			
NGO0513	489659–490129	156	NS			
NGO0514	490130–490462	110	NS			
NGO0515	490591–490824	77	NS			
NGO0516	490832–491179	115	PemK			
NGO0517	491179–491415	78	PemI	(pfam SpoVT AbrB family)		
NGO0518	491622–492161	114	hypothetical protein	NMB0912 protein [*N. meningitidis *MC58] 32, 45% on 53		1649
NGO0519	492162–492506	120	NS			1650
NGO0520	492680–492990	103	NS			
NGO0521	493004–493153	49	hypothetical protein	NMB0989 protein [*N. meningitidis *MC58] 55, 61% on 47		
NGO0522	493183–496230	1015	tail length tape	JL00p82 [Phage phi JL001 measure protein 29, 50% on 407 putative tail component [Z6034] protein cryptic prophage CP-933P [*E. coli*] 27, 47% on 259		
NGO0523	496290–496769	159	hypothetical protein	NMB0899 [*N. meningitidis *MC58] 100, 100% on 118	1095	
NGO0524	497111–498100	329	integrase	[*Haemophilus influenzae *R2866] (pfam phage integrase family 49, 62% on 200		1613

* No significant homology with any gene in the database

### Modular genome organization of phage NgoΦ1

The DNA sequence of phage NgoΦ1 contains 62 CDSs that we annotated as phage related genes (Fig. 2). A large number of the CDSs show significant homology to other known phage genes (Table 3). Some of the CDSs encode putative proteins with no current functional annotation. Based on available data, sequence similarity, and domain and motif searches, information indicating for modular organization of the genome was obtained. Both ends of the phage DNA encoded two bacteriophage P4-like integrases belonging to pfam 00589 family (NGO0462 and NGO0524) with no homology between either of them. The P4 integrase mediates integrative and site-specific recombination between two attachment sites, located on the phage genome and the bacterial chromosome. NGO0462, encoding the first of these integrases, is preceded by the sequence encoding serine tRNA (NGO0461) and a 151 bp noncoding region. Similarly, a noncoding region is also present downstream of the last phage gene (NGO0524). We were unable to identify any repetitive sequences at either end of the integrated phage DNA.

Analysis of the CDSs encoded by this phage allowed us to clearly identify two blocks of genes encoding structural and assembly proteins. The longer block spanning from NGO0494 to NGO0505 includes such genes as NGO0494 and NGO0495 which encode small and large terminase subunits respectively, NGO0496 which encodes phage portal protein and NGO0510, which encodes a phage tail protein. The smaller block of structural genes is located at the end of phage genome (NGO0522 and NGO0523) and encodes the tail protein. The remaining CDSs encode either proteins of unknown function or other structural proteins. It is interesting that gene NGO0509, which interrupts the larger block, belongs to the transcriptional regulator family of genes. The regulatory modules include one well defined region (NGO0474 to NGO0483) with NGO0479 encoding a protein similar to cI-like repressor protein. Two less defined regulatory modules are located in the region between NGO0515-0517 and in the region of NGO0464. The main regulatory module are predicted to encode not only cI repressor but also other proteins that show some homology to phage and cell transcriptional regulatory proteins, (NGO0474, NGO0477, and NGO0478), or the presence of a domain specific for regulatory protein (NGO0483). NGO0479 contains well defined helix-turn-helix motif (E value 6^-4^) between I^30 ^and F^75 ^and high scoring (E value 5^-6^) from V^147 ^to D^219 ^peptidase family S24. These motifs are present in DNA binding proteins, including the cro and cI proteins of phage lambda. NGO0477 also contained a helix-turn-helix motif (E value: 4^-10^).

The special control region includes two CDSs, NGO0516 and NGO0517, which encode PemK and PemI-like proteins. The pemI-pemK system is an addiction module present on plasmid R100 that helps to maintain the plasmid by post-segregational killing of *E. coli *cells that have lost the plasmid [19,20]. PemK, the toxin encoded by the *pemI-pemK *addiction module, inhibits protein synthesis in an *E. coli *cell-free system, whereas the addition of PemI, the antitoxin against PemK, allows for the resumption of protein synthesis. These systems are also known to operate by stabilizing the presence of autonomously replicating prophages, like P1 [21].

The third module includes genes that play a role in the replication of the phage genome. This module includes two well defined CDSs, NGO0484 and NGO0485, and a third CDS, NGO0469, located to left end of the genome. NGO0485 encodes a helicase with high homology to the phage P1 gene *ban *or gp12 of *Salmonella typhimurium *bacteriophage ST64T (Table 3). All these proteins belong to pfam DnaB, DnaB-C family. NGO0484 shows high homology to a DNA replication protein of bacteriophage AAphi23 as well as other phages. Finally, the NGO0469 probably encodes the primase.

### Modular genome organization of phage NgoΦ2

The sequence of NgoΦ2 shows a modular organization and some regions possess almost 100% homology with the DNA sequence of NgoΦ1. The overall orientation of the DNA sequence and the localization of the genes in NgoΦ2 are in the opposite orientation relative to NgoΦ1. We were also unable to find repetitive DNA sequences at the end of the DNA sequence of the CDSs encoding the integrases (Fig. 2, Table 4). The regions of homology cover the sequences from the left end of both phages (except of first 1600 bp) up to the beginning of the gene encoding presumptive recombination module, and the module of the structural genes, with the exception of the module controlling the transcription (Fig. 2) in NgoΦ1 (NGO0475 to NGO0480). This suggests that while these two DNA sequences represent two different phages, they share homology between genes encoding the replication machinery but do not share homology between the genes responsible for the control of gene expression, maintenance of the state of lysogeny (the genes encoding presumptive repressors; NGO0479 in NgoΦ1 and NGO1116 in NgoΦ2), in the recombination module and in the structural modules of both phages. The putative repressor (NGO1116) contains a well defined helix-turn-helix motif (E value 8^-04^) between M^1 ^and L^40 ^and high scoring (E value 7^-04^) peptidase family S24 motif from P^123 ^to D^200^. However, the putative antirepressor (NGO1085) is located at the end of the genome, a long distance from the repressor. NGO1085 shows high homology to some of the phage antirepressors, like that of bacteriophage Aaphi23 or *E. coli *prophage CP-933N.

**Table 4:** Properties of the NgoΦ2 CDS' sequences

NgoΦ2 CDSs	Genome position	Length (aa)	Putative function	Significant matches to proteins in GenBank (Scores represent filtered BLASTP % identities over stated number of amino acid) CDSs
NGO1085	1044447–1045292	281	prophage antirepressor protein 933N], 37, 56% on 20	Ant [Phage Aaphi23], 41, 56% on 258 (pfam02498, Bro N family) [*E. coli *prop CP933N], 37, 56% on 20
NGO1087	1045494–1046174	226	NS	
NGO1088	1046153–1046638	161	hypothetical protein	F116p45 [*P. aeruginosa *phage F116] 31, 42% on 148
NGO1089	1046644–104711	157	phage structural protein	F116p44 [*P. aeruginosa *phage F116] 42% on 140
NGO1090	1047173–1048468	431	phage structural protein	F116p43 [*P. aeruginosa *phage F116] 56, 72% on 415
NGO1091	1048489–1048887	132	hypothetical protein	F116p42 [*P. aeruginosa *phage F116] 34, 49% on 141
NGO1092	1049094–1055027	1977	possible DNA methylase	[*Sinorhizobium meliloti *phage PBC5] 33, 52% on 584
NGO1093	1055027–1056445	472	NS	[*S. meliloti *phage PBC5] 33, 52% on 584 hypothetical protein F116 p60
NGO1094	1056442–1057638	398	NS	
NGO1096	1058422–1058637	71	NS	
NGO1097	1058674–106026	750	phage portal protein	F116p40 [*P. aeruginosa *phage F116] 58, 71% on 756
NGO1098	1060926–1062200	424	phage terminase large subunit Terminase 3 family	ZMO0379 [*Zymomonas mobilis *subsp.mobilis ZM4]phage terminase large subunit 35, 50% on 397
NGO1099	1062181–1062423	80	NS	[*H. influenzae *RdKW20] 44, 67% on 168
NGO1100	1062478–1062720	80	prophage terminase small subunit	STY1046 [*S. enterica *subsp. enterica serovar Typhi] small subunit 33, 57% on 45
NGO1101	1062720–1063034	104	hypothetical protein	F116p37 [*P. aeruginosa *phage F116] 38, 59% on 81
NGO1102	1063207–1063725	172	endonuclease of the HNH family	17R [*Xanthomonas oryzae *phage endonuclease Xp10] 41, 53% on 167 [*S. thermophilus *phage ST3] 47, 55% on 85
NGO1103	1063716–1064099	127	protein Nin B	HK022 p48 [*Enterobacteria *phage HK022]30, 50% on 121, 933Wp33 [Phage 933W] 27, 49% on 99
NGO1104	1064096–1064401	101	hypothetical protein	F116 p35 [*P. aeruginosa *F116] 42, 57% on 98 pfam DUF 1364 CDS-136 [*S. typhimurium *phage ST64T] 53, 66% on 30
NGO1105	1064373–1064993	206	NS	
NGO1107	1065246–1065614	122	hypothetical protein	NMB1116 [*N. meningitidis*] 88, 96% on 27
NGO1115	1069893–1070081	62	NS	
NGO1116	1070260–1070907	215	transcriptional regulator	NMB0910 [*N. meningitidis *regulator MC58] 83, 87% on 215, F116p29 [*P. aeruginosa *phage F116] 39, 54% on 157 putative CI protein cI [Phage Aaphi23]37, 54% on 191, cI [*Pseudomonas *phage D3]30, 44% on 218
NGO1117	1071067–1071606	179	hypothetical protein	CDS21 [Phage bIL311] GepA [phage-like] 37, 53% on 135, SpyM3_1207 [*S. pyogenes *phage 315.4] 40, 66% on 71
NGO1118	1071607–1071966	119	NS	
NGO1119	1071983–1072201	72	NS	
NGO1132	1078087–1078281	64	hypothetical protein	33 [*Enterobacteria *phage epsilon15] 68% on 44

The structural and assembly module, extending from NGO1105 to NGO1086, contains the genes encoding the large and small terminase proteins (NGO01098 and NGO01101 respectively) and the portal protein (NGO01097). The structural module contains one very large protein (1977 aa) encoded by NGO1092, whose C-terminal part shows some homology to DNA methyltransferases. NGO1085 would seem to encode a protein with homology to phage antirepressors. While the structural module of NgoΦ2 (NGO1102 to NGO1186) shows high identity with *P. aeruginosa *phage F116, the structural block of NgoΦ1 does not show high identity with any particular phage sequence.

### Modular genome organization of phage NgoΦ3

The DNA sequence of NgoΦ3 is much smaller than NgoΦ1 and NgoΦ2 and is disrupted by the insertion of the DNA sequence of ssDNA phage NgoΦ9. The first part of the sequence (16021 bp) encodes 28 CDSs while the second part of (2980 bp) encodes only four CDSs. The genome sequence starting with NGO1613, encodes a P-22-like integrase but without homology to the integrase encoded by NgoΦ1. The majority of the DNA sequence shows homology to DNA sequence of NgoΦ1 and NgoΦ2. Two regions without homology include: (i) NGO0475 – NGO0409 responsible for the maintenance of lysogenic state (encoding a repressor) and (ii) NGO0482-NGO0487, genes responsible for DNA replication. There is also lack of the NgoΦ1 DNA region encoding PemK-PemI proteins. However, the most important difference between the genomic sequences of NgoΦ3 and NgoΦ1 and NgoΦ2 is the lack of CDSs encoding the structural protein (structural-assembly module). The second part of the genome of NgoΦ3 encodes the antirepressor protein (NGO1652) which is identical with NGO1085 encoded by the phage NgoΦ2.

### Modular genome organization of phages NgoΦ4 and NgoΦ5

Phage DNA sequences corresponding to NgoΦ4 and NgoΦ5 (Fig. 2) do not share any homology to each other, or with NgoΦ1, NgoΦ2 and NgoΦ3. Analysis of the homology of the CDSs encoded by these two sequences indicates that neither of them represents a complete phage genome (see Tables 5 and 6). The DNA sequence of NgoΦ4 contains the genes encoding only the presumptive proteins engaged in the recombination, control of transcription and integration of DNA. NGO1000 and NGO1001 contain motifs typically characteristic for the RecBC family exonuclease protein, while NGO1020 contains the core domain characteristic for pfam 00665, Rve integrase. This family of genes is responsible for integration of the viral genome into the host chromosome. NGO1007 encodes protein with high homology to the transcription regulator proteins encoded by several phages, among them the Stx converting phages.

**Table 5:** Properties of the NgoΦ3 CDS' sequences

NgoΦ3 CDSs	Genome position	Length (aa)	Putative function	Significant matches to proteins in GeneBank (Scores represent filtered BLASTP % identities over stated number of amino acid)
NGO1613	1592225–1593043	384	integrase	[*Xylella fastidiosa *Dixon] (phage integrase family) 0524 35,52% on,367, [*Pseudomonas syringe *tomato str. DC3000] 24,38% on 340, int [*Azoarcus sp. *EbN1] 25,42% on 365
NGO1614	1595199–1595750	88	NS	
NGO1625	1596026–1596241	138	NS	
NGO1626	1583028–1584182	215	conserved hypothetical protein	CDS21 [Bacteriophage bIL311] (pfam GepA,) 29,48% on 116
NGO1627	1584232–1584498	133	hypothetical protein	HP2p14 protein [*Haemophilus *phage (pfam UPFO150 family) HP2] 34,54% on 129
NGO1628	1590177–1590593	60	conserved hypothetical protein	[HP1p18] protein [*Haemophilus *(COG1724) phage HP1] 60,74% on 51
NGO1629	1590602–1591249	272	NS	
NGO1634	1591369–1591770	183	hypothetical protein	hypothetical protein [P27p17] [Bacteriophage P27] 30,51% on 147 replication protein [SfVp87] [*Shigella flexneri *bacteriophage V] 32,54% on 87
NGO1635	1591873–1592055	71	NS	
NGO1636	1596419–1597036	205	DnaC-like protein	[STM2625] protein [Phage Gifsy-1] (pfam IstB family) 37,51% on 201, replication protein DnaC [Bacteriophage P27] 37,56% on 192
NGO1637	1597106–1597261	51	NS	
NGO1639	1597884–1598387	167	hypothetical protein	NMB1116 *[Neisseria meningitidis *MC58] 88,96% on 27
NGO1651	1608278–1608616	113	Neisseria-specific protein, uncharacterized	
NGO1652	1609818–1608823	332	putative antirepressor	protein Ant [Bacteriophage (pfam Bro-N family) 1085 Aaphi23]

**Table 6:** Properties of the NgoΦ4 CDS' sequences

NgoΦ4 CDSs	Genome position	Length (aa)	Putative function	Significant matches to proteins in GeneBank (Scores represent filtered BLASTP % identities over stated number of amino acid)
NGO1000	982212–984008	75	RecB family exonuclease	
NGO1001	973188–973673	161	RecB family exonuclease	
NGO1002	973680–974048	122	transcriptional factor	[*Exiguobacterium sp*. 255-15] antiterminator 27,53% on 77 (traA) [Lactococcus lactis] 20,46% on 100
NGO1003	977880–978314	184	hypothetical protein	BH0339 [*Bacillus halodurans *C-125] (pfam DUF694 family) 36,55% on 180
NGO1004	978515–978889	119	NS	
NGO1005	978886–979764	288	NS	
NGO1006	980160–980540	116	possible transposase	[NMA1601b][*Neisseria meningitidis *Z2491 79,83% on 43
NGO1007	980749–981042	69	hypothetical protein	VT2-Sap81 [Bacteriophage VT2-Sa] (pfam zf-*dskA-traR *family) 42,55%on69, phi4795p07 [Phage phi4795]40,53% on 69
NGO1008	981023–981313	96	Neisseria-specific protein	NMB1087 [*Neisseria meningitidis *MC58] 44,56% on 67
NGO1009	974075–974617	95	Neisseria-specific protein	NMB1014 [*Neisseria meningitidis *MC58] 93,94% on 75
NGO1010	975275–976141	144	conserved hypothetical protein	*Pseudomonas aeruginosa *32,51% on 137
NGO1011	976191–976541	124	NS	
NGO1012	976976–977185	292	NS	
NGO1013	977473–977760	126	transcriptional regulator	[NMB1007] [*Neisseria meningitidis *pfam HTH 3 family phage repressor MC58] 60,72% on 126
NGO1014	972837–973064	97	hypothetical protein	NMB1005 [*Neisseria meningitidis *MC58] 79,84% on 73 B3CDS17 [Bacteriophage B3] 46,59% on 47
NGO1015	974783–975142	96	Neisseria-specific protein	NMB1003 [Neisseria meningitidis MC58] 77,90% on 95 hypothetical protein B3CDS14 [Bacteriophage B3]32,61% on 90
NGO1020	977178–977468	598	putative transposase	[Bacteriophage B3] 43,61% on 585 (pfam rve family) integrase protein [*Salmonella typhimurium *LT2] 25,41% on 383

Among the CDSs encoded by the DNA sequence of NgoΦ5 are ones that show high homology to structural and assembly proteins of different phages (see Table 7) but lack such homology to the small and large terminase subunits and the portal proteins. At the end of DNA sequence are genes encoding a putative repressor and transposase. The organization of these genes resemble the pyocin R2 and F2 gene cluster present in *P aeruginosa*, which are phage tails that have been evolutionarily specialized as bacteriocins [22].

**Table 7:** Properties of the NgoΦ5 CDS' sequences

NgoΦ5 CDSs	Genome position	Length (aa)	Putative function	Significant matches to proteins in GeneBank (Scores represent filtered BLASTP % identities over stated number of amino acid)
NGO0720	721256–721459	67	hypothetical protein	NMB1387 [*Neisseria meningitidis *MC58] 99,99% on 44
NGO0721	729286–729570	337	hypothetical protein	NMA1603 [*Neisseria meningitidis *Z2491] 72,76% on 173
NGO0722	721459–722472	46	hypothetical protein	NMA1602 [*Neisseria meningitidis *Z2491] 94,97% on 36
NGO0723	722517–722657	82	hypothetical protein	NMA1216 [*Neisseria meningitidis *Z2491] 94, 100% on 37 HP2p14 [Haemophilus phage HP2] 31, 61% on 2
NGO0724	72289–723144	40	hypothetical protein	NMA1216 [*Neisseria meningitidis *Z2491] 97, 97% on 34
NGO0725	723086–723208	102	hypothetical protein	NMB1116 [*Neisseria meningitidis *MC58] 54,62% on 90
NGO0727	723158–723466	182	phage baseplate assembly	[o0965] [*Escherichia coli *CFT073] (pfam Baseplate J family) 54, 67% on 146, gpJ [*Enterobacteria *phage P2] 48, 61% on 146
NGO0728	726767–727315	300	phage baseplate assembly protein	CDS17 [bacteriophage phi CTX] (pfam Phage base V family) gpW [Enterobacteria phage P2] (pfam GPW gp25 family 37,57% on 90
NGO0729	727336–728238	70	putative cI-like repressor	[*Streptococcus pyogenes *phage 315.3] 45,67% on 31
NGO0730	728344–728556	217	IS1016C2 transposase	[*Neisseria meningitidis *MC58] 78, 82% on 217 protein V6-*Haemophilus influenzae *55,69% on 175
NGO0731	728611–729264	94	hypothetical protein	NMA1219 [*Neisseria meningitidis *Z2491] 97, 97% on 82

### Distribution of the dsDNA prophage sequences among the *N. gonorrhoeae *strains

**Figure 3:**
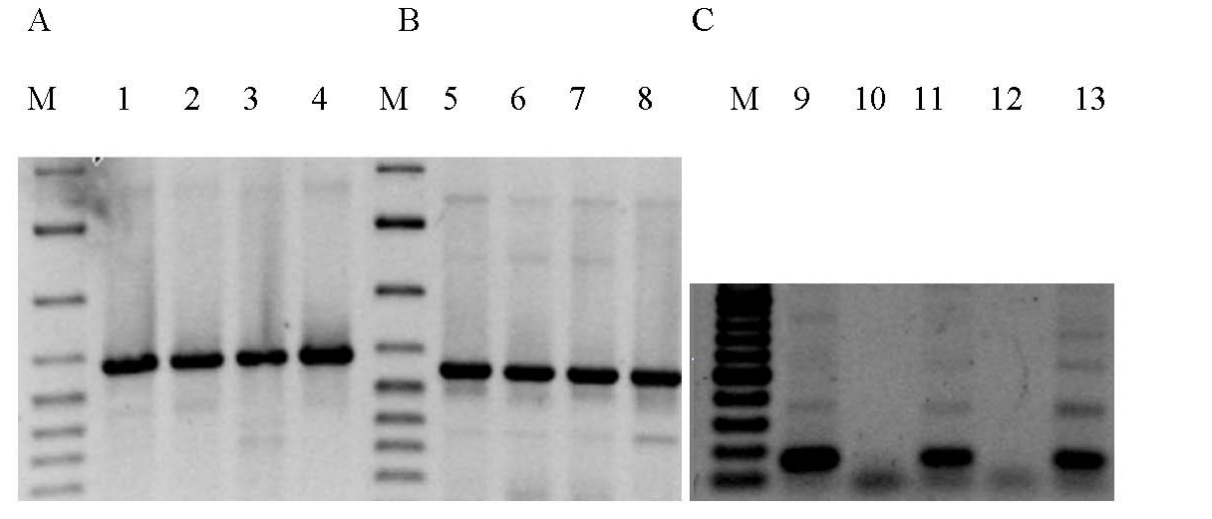
**PCR detection of the presence of dsDNA prophage sequences NgoΦ1 and NgoΦ2 sequences in different strains of *Neisseria***. A. Detection of the prophage NgoΦ1 CDS495 sequence encoding putative large terminase subunit, B. Detection of the prophage NgoΦ2 CDS1098 sequence encoding putative large terminase subunit, C. Detection of the CDS448 encoding putative holin of NgoΦ1/NgoΦ2. Lanes 1, 5, 9, *N. gonorrhoeae *FA1090, lanes 2, 6, 11; *N*. *gonorrhoeae *MS11, Lanes 3, 7, 13; *N. gonorrhoeae *WR220, lanes 4, 8, 12; *N. gonorrhoeae *WR302, Lane 10 *N*. *gonorrhoeae *1291. M, molecular weight markers.

**Figure 4:**
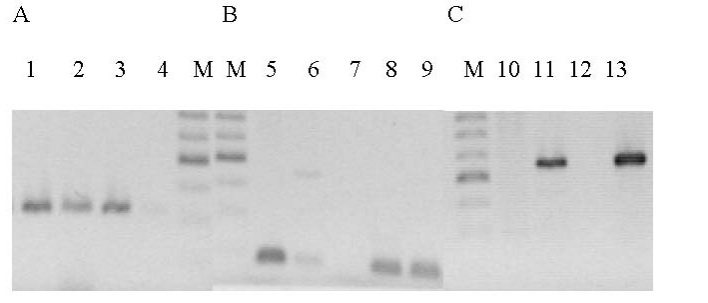
**PCR detection of the presence of dsDNA prophage NgoΦ3 – NgoΦ5 sequences in different strains of *Neisseria***. A. Detection of the prophage NgoΦ4CDS1013 encoding the putative repressor protein, B. Detection of the prophage NgoΦ5 CDS729 encoding the putative repressor, C. Detection of the CDS1636 encoding putative *dnaC *gene of NgoΦ3. Lanes 1, 5, 13; *N. gonorrhoeae *FA1090, lanes 2, 6; *N. gonorrhoeae *WR302, Lanes 3, 8; 11, *N. gonorrhoeae *MS11, Lanes 4,7,10; *N*. *gonorrhoeae *WR220, Lanes 9, 12, *Neisseria gonorrhoeae *1291. M, molecular weight markers

In *N. meningitidis *the phages homologous to ssDNA phages are present predominantly in the hypervirulent isolates [16]. On the other hand, the Mu-like prophage sequences are present in all *N. meningitidis *strains [15]. We tested for the presence of the dsDNA prophage sequences in the chromosome of *N. gonorrhoeae *FA1090 and other *Neisseria *strains by the formation of the PCR products specific for the NgoΦ1 and NgoΦ2 prophages CDSs encoding the large terminase subunit, holin and the repressor of NgoΦ2 prophage. The results presented in Fig. 3 indicate that the amplicons obtained from the four *N. gonorrhoeae *strains tested were of the predicted molecular mass as the PCR amplicon generated from strain FA1090 suggesting that in all cases the same gene was present. DNA sequence analysis of the amplicons confirmed that the amplicons contained the predicted gene. When *N. meningitidis *or *N. lactamica *genomic DNA were tested, only non-specific PCR products were formed, suggesting the lack of these genes in these species (data not shown). When we tested for the formation of the PCR product corresponding to NGO0488 encoding the putative holin, products were formed with DNA isolated from strains FA1090, MS11 and WR220, but not with WR302 or 1291 DNA (Fig. 3C). Since the sequence corresponding to NGO0488 is identical with the sequence encoding NGO1106 present in NgoΦ2, the lack of the proper PCR product indicates the lack of a complete NgoΦ1 and NgoΦ2 phage genome in these two strains, or that there is CDS encoding a different holin. Similarly, when we used the primers specific for the NGO1116 (present in NgoΦ2), encoding putative repressor protein, the correct PCR product was formed with *N. gonorrhoeae *FA1090 and 1291 chromosomal DNAs but not with WR220 genomic DNA (data not shown). Using the PCR and primers specific for NgoΦ3-NgoΦ5, we found that the genes were variably present in the strains that we tested (Fig. 4).

### Biological activity of the prophage repressor genes

The maintenance of the lysogenic state is most often maintained by the action of a repressor gene. Functionally, the repressor is silencing the activity of most of prophage genes. However, the expression of several genes, whose transcription is constitutive, is independent of its control. Recently it was shown that host genes can be regulated by phage repressors active in the lysogenic cells [23]. If this were true for the dsDNA prophages integrated into *N. gonorrhoeae *FA1090, we would expect that the phage repressors could influence the expression of cellular genes. We tested for their biological activity in *E. coli*.

**Figure 5:**
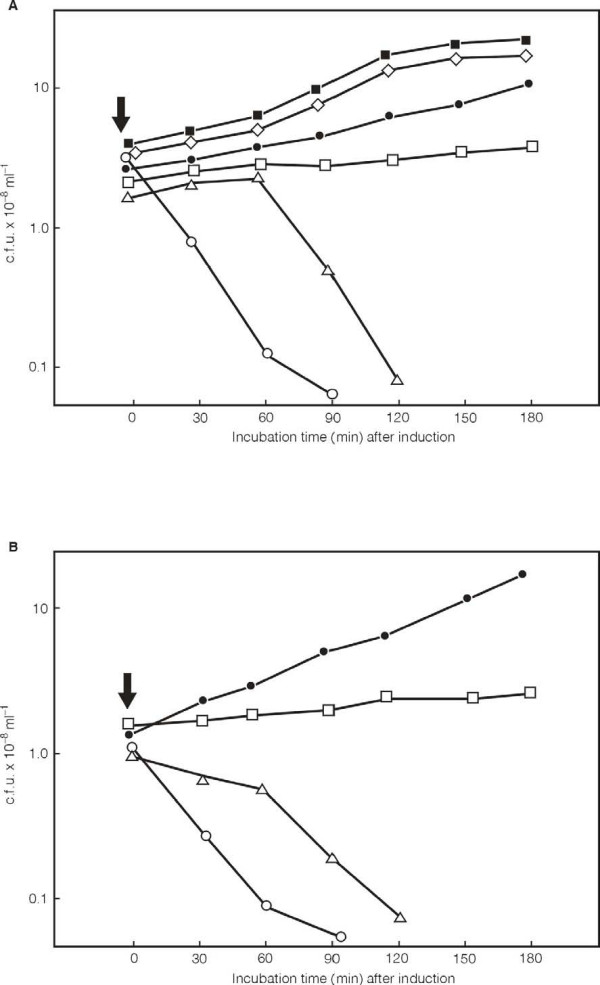
**Influence of expression of the NgoΦ1 and NgoΦ2 putative promoters on the growth of *E. coli *Top10 cells as measured by the change of (CFU/ml)**. The overnight culture of the *E. coli *Top10 cells carrying cloned CDS479 gene (A) or CDS1116 (B) on the plasmid pMPMK6 grown in LB was diluted 1:50 into 50 ml of the fresh medium and grown at 37°C until the OD_650 _was equal of about 0.8. At that time the culture was divided and the expression of the cloned gene was started by the addition of the different concentration of arabinose. (A) Symbols: (filled circles), *E. coli *Top10 (pMPMK6::cds479) not induced; (open squares), *E. coli *Top10 (pMPMK6::cds479) induced with 0.001% arabinose; (open triangles), *E. coli *Top10 (pMPMK6::cds479) induced with 0.01% arabinose; (open circles), *E. coli *Top10 (pMPMK6::cds479) cells induced with 0.1% arabinose; (filled squares), *E. coli *Top10 (pMPMK6) cells not induced; (open diamonds), *E. coli *Top10 (pMPMK6) cells induced with 0.1% arabinose. (B) Symbols: (filled circles), *E. coli *Top10 (pMPMK6::cds1116) not induced; (open squares), *E. coli *Top10 (pMPMK6::cds1116) induced with 0.001% arabinose; (open triangles) *E. coli *Top10 (pMPMK6::cds1116) induced with 0.01% arabinose; (open circles) *E. coli *Top10 (pMPMK6::cds1116) cells induced with 0.1% arabinose. The arrow shows the time when arabinose was added.

The analysis of the NgoΦ1 and NgoΦ2 genomes allowed us to tentatively identify the repressor genes in both prophages as the CDSs NGO0479 and NGO1116 (Fig. 2, Table 4 and Table 5). Both genes carrying only the coding sequences were cloned in *E. coli *in pMPMK6 Ω [24], where expression of the gene was placed under control of the pBAD promoter. We expressed the repressor and tested it for its ability to influence the growth of *E. coli *and for its ability to inhibit the propagation of phage λ. The first experiment showed that even without induction, the presence in *E. coli *of a plasmid that encodes the repressors inhibits the growth of the host *E. coli *strain. The induction of NGO0479 by arabinose resulted in cell death (Fig. 5A) but not cell lysis (data not shown). Loss of viability seemed to be dependent on the level of protein expression, as maximal induction of the pBAD promoter (0.1% arabinose) produced a rapid and almost immediate decrease in cell viability: lower amounts of induction produced reduced killing, or simple growth inhibition. A similar effect was also observed after induction of the expression of NGO1116 (Fig. 5B). From this data we concluded that cell death results from the expression of the cloned phage genes.

**Figure 6:**
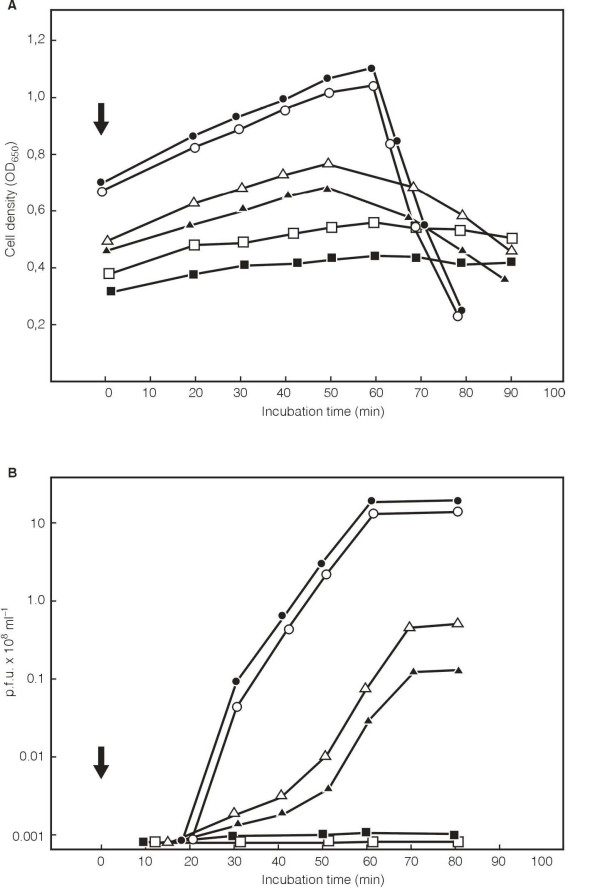
**Influence of expression of the NgoΦ1 prophage CDS495 and NgoΦ2 CDS1116 encoding the putative repressors on production of the phage λ. *E. coli *3102 (λ_cI857_)**. Cells carrying cloned CDS479 or CDS1116 were grown overnight at 30°C and then diluted 1:50 in the fresh LB medium. The growth was continued for 3 hr at 30°C. At that time the culture was divided and the expression of the cloned gene was induced by the addition of the arabinose to a final concentration of 0.001%. After 45 min of growth at 30°C the cultures were heat induced at 43°C for 15 min and then the growth was continued at 37°C. The samples were withdrawn at different times and the optical density (A) and (B) the free phage titer (after treatment with chloroform, 10% final concentration for 15 min) were determined. Symbols: (filled circles) *E. coli *3102 (λ_cI857_) (pMPMK6) cells no arabinose was added; (open circles) *E. coli *3102 (λ_cI857_) (pMPMK6) cells induced with arabinose; (open triangles), *E. coli *3102 (λ_cI857_) (pMPMK6::cds1116) no arabinose added; (filled triangles) *E. coli *3102 (λ_cI857_) (pMPMK6::cds1116) cells induced with arabinose; (open squares), *E. coli *3102 (λ_cI857_) (pMPMK6::cds479) no arabinose added; and (filled squares), *E. coli *3102 (λ_cI857_) (pMPMK6::cds479) cells induced with arabinose. The arrow shows the time when the heat induced cultures were transferred into 37°C.

To test if cell death was the result of the influence of the expression of chromosomally encoded genes, the influence of the expression of the two repressors on propagation of the phage λ was tested. Plasmids encoding both repressors were introduced into *E. coli *3102, a strain that is lysogenic with λ_cI*ts*857_. The induction of the NGO0479 by the presence of 0.001% arabinose, a concentration that induced the expression of some repressor and inhibited the growth of *E. coli*, did not result in significant death of the culture. Heat induction of the λ phage did not induce cell lysis but blocked completely production of the progeny phage (Fig. 6). On the other hand, the induction of the NGO1116 and the heat induction of phage λ resulted in some cells lysis (data not shown) and the 10 times lower phage production than without the presence of the NGO1116 gene product (Fig. 6). This data indicates that very low level of expression of both repressors influences the propagation of the λ phage.

Since the expression of the phage repressors affected the growth of *E. coli*, we hypothesized that it was influencing the expression of as yet undefined genes. To further support this hypothesis, we tested the influence of the NGO1116 product on the expression of different HP1 phage promoters. We constructed plasmids carrying transcriptional fusions in which the xylE gene was expressed from the different HP1 phage promoters. The data in Table 8 indicate that both promoters are active in *E. coli*, as evidenced by the presence of XylE activity in supernatants obtained from host cells grown in the absence of arabinose. When arabinose was added, the XylE activity found in supernatants isolated from these strains indicates that P_LHP1 _promoter but not P_RHP1 _activity is inhibited. This data indicates that the NGO1116 gene product can differentially repress promoter activity, and suggests that its expression in the gonococcus could silence prophage gene expression in the *N. gonorrhoeae *genome.

**Table 8:** The influence of expression of CDS1116 on the different promoter activity.

Strain	XylE activity (U)^a^
*E. coli *Top10 (pMPMT4::CDS1116, pMPMK6::P_RHP1_) - ara	22.3 +/- 10.2
*E. coli *Top10 (pMPMT4::CDS1116, pMPMK6::P_RHP1_) + ara	20 +/- 9.5
*E. coli *Top10 (pMPMT4::CDS1116, pMPMK6::P_LHP1_) - ara	268.2 +/- 33.1
*E. coli *Top10 (pMPMT4::CDS1116, pMPMK6::P_LHP1_) + ara	20.0 +/- 9.2

^a^One unit of XylE activity corresponds to the formation of 1 nmol of 2-hydroxymuconic semialdehyde per min at 22°C.

### The putative holin proteins

Most bacteriophage must lyse their host cells to liberate the progeny virions. The decision of when to terminate the infection and to lyse the host is the only major decision made in the vegetative cycle [25]. Thus, if the prophage sequences present in the genome of *N. gonorrhoeae *are able to excise from the bacterial genome and produce progeny, they should encode a lytic system. Phages with double-stranded nucleic acid genomes use the "holin-endolysin" strategy [23]. In this scheme, the phage encodes murein-degrading enzyme, an endolysin, and a second membrane-embedded protein, holin, which serves to activate the endolysin at the defined time. Holins are small membrane proteins that accumulate in the cytoplasmic membrane of the host. The holins are represented by more than 250 members belonging to 50 gene families with no recognizable detectable sequence similarity [25]. The common property of all holins is the presence of one or more trans-membrane domains [25], which allows for their identification in the phage sequences. Our analysis of the proteins encoded by the prophage NgoΦ1-NgoΦ3 sequences suggested that the NGO0488, NGO1106 and NGO1622 could potentially encode a holin. NGO0488 and 1106 are identical and encode for a protein of 49 amino acids, while NGO1622 encodes for a protein of 91 amino acids. These proteins do not showhomology to any other proteins and each possesses one TM domain. To test whether the NgoΦ1 putative holin can act as a true holin, we tested to see if this CDS could complement mutations in the phage λ *S *gene. For controlled expression of NGO0488, this gene was inserted downstream of the pBAD promoter of pMPMK6 [24] resulting in the construct pMPMK6*hol*. The *E. coli *MM 294 cells (λ*c*I_857 _*S*am7), carrying the plasmid pMPMK6::*hol *were grown at 30°C to the optical density of OD_600 _of 0.6. At that time, the heat shock was carried out (45°C for 20 min) and arabinose was added to a final concentration of 1.0%. In the presence of *hol *gene of NgoΦ1 phage, even without its induction, the cells grew very slowly. We were unable to detect the lysis of these cells after induction by arabinose (data not shown). Thus, in these experiments the uninduced level of holin was sufficient to inhibit the growth of *E. coli *cells, carrying an uninduced λ prophage.

**Figure 7:**
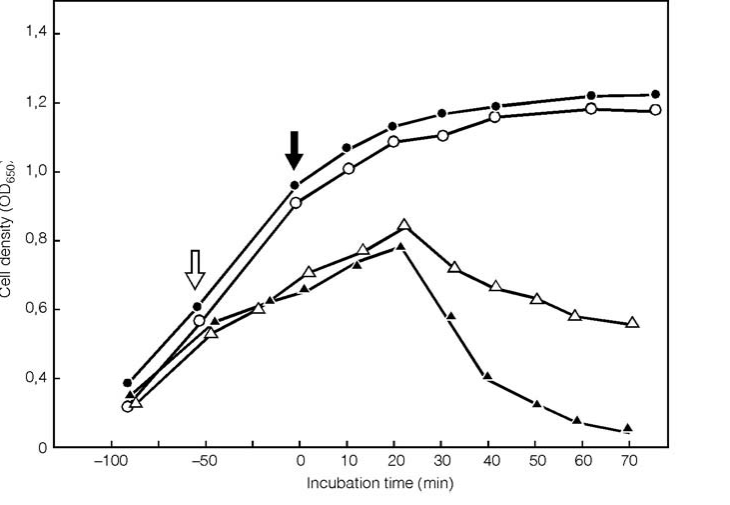
**Complementation of the λ*S *am7 mutant with the CDS488 of NgoΦ1**. 50 ml of LB medium containing the 1% glucose was inoculated with 1 ml of overnight culture of *E. coli *MM294 λ111 (*cI*857 *S*am7) carrying pMPMK6::cds448) grown in the LB medium in the presence of 1% glucose at 30°C. After 2.5 h of growth at 30°C the culture was centrifuged at 5000 rpm for 10 min, and the cells suspended in the 50 ml of fresh LB medium without glucose. The λ111 (*cI*857 *S*am7) was induced by incubation of the culture at 44°C for 15 min and the expression of the CDS488 gene was (or not) induced by the addition of arabinose to a final concentration of 0.001%. The white arrow shows the time of CDS488 induction, the black arrow shows the time of heat induction. Symbols: (filled circles), *E. coli *MM294 λ111 (*cI*857 *S*am7) carrying pMPMK6), no arabinose induction; (open circles), *E. coli *MM294 λ111 (*cI*857 *S*am7) carrying pMPMK6, with arabinose induction; (open triangles), *E. coli *MM294 λ111 (*cI*857 *S*am7) carrying pMPMK6::cds488, no induction with arabinose; and (filled triangles), *E. coli *MM294 λ111 (*cI*857 *S*am7) carrying pMPMK6::cds488, induction with arabinose.

In order to assess whether Hol resembles S and other holins-particularly with respect to the salutary triggering phenotype that characterizes holin functions, conditions which allow for even tighter regulation of the pBAD promoter but allow for a high level of induction were needed. The experiments were then carried out in a similar way, but the culture, prior to heat inactivation and arabinose induction, was grown in the presence of 1% glucose, to further block the pBAD promoter through catabolic repression. The growth in the presence of glucose was still slower than the growth of *E. coli *MM 294 cells (λ*c*I_857 _*S*am7) without the presence of *hol *gene. However the induction of *hol *gene resulted in abrupt lysis of entire culture, with the onset of lysis occurring about 20 min after induction (Fig. 7). Thus, like the well-studied λ holin S, and other phage holins, the holin of NgoΦ1 causes abrupt lysis of *E. coli *cells at a defined time.

### Presence of the dsDNA phages in *N. gonorrhoeae *supernatants

To detect the presence of phage DNA in phage particles released from the cells during growth, the same method was used as described previously for the detection of the filamentous ssDNA phages in *N. meningitidis *cultures [16]. In this method, the presumptive phage particles are precipitated with PEG and the precipitate intensively treated with DNase and RNAse to remove contaminating bacterial genomic DNA and RNA. The DNA is then isolated from the phage and used as a template in PCR reactions. The presence of the particular phage DNA sequence in such phage preparations means that the prophage genome must be excised from the bacterial genome, and after a replication cycle, packaged into the phage particles and released from the cells.

**Figure 8:**
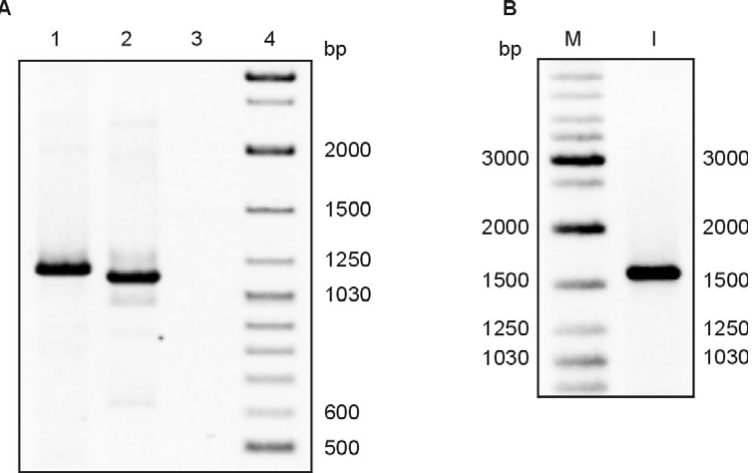
**Detection of the extracellular dsDNA of NgoΦ1 and NgoΦ2 phages of *N. gonorrhoeae***. PCR amplification was performed on DNA from phage preparation to detect the presence of the DNA sequence corresponding to large terminase encoded by CDS488 (Lane A1) or CDS1116 (Lane A2). In the control experiment the PCR product corresponding to the chromosomal DNA sequence of *N. gonorrhoeae *FA1090 encoding the lpt3 gene was not detected (Lane A3) in the phage preparation while the same PCR product was formed using the chromosomal DNA as a substrate (Lane B1).

In similar experiments, the presence of the dsDNA isolated from the crude phage preparation obtained from the *N. gonorrhoeae *FA1090 cultures was tested. Using primers specific for NgoΦ1 (NGO0495) we were able to show the formation of predicted PCR product (Fig. 8). To show that the phage preparation does not contain the bacterial genomic DNA released from the growing bacterial cells, we performed a PCR amplification for the chromosomally encoded gene, *lpt3*. The data (Fig. 8) indicate that an amplicon was formed when chromosomal DNA was used as template, but not when phage DNA was employed. This result indicates that in *N. gonorrhoeae *FA1090 the prophage sequences can be excised from the chromosome, replicate and form phage particles that are released from the bacterial cells. We also tested the possibility that such phage can form plaques on *N. subflava, N. lactamica *or *N. cinerea *cells, but without success (data not presented).

**Figure 9:**
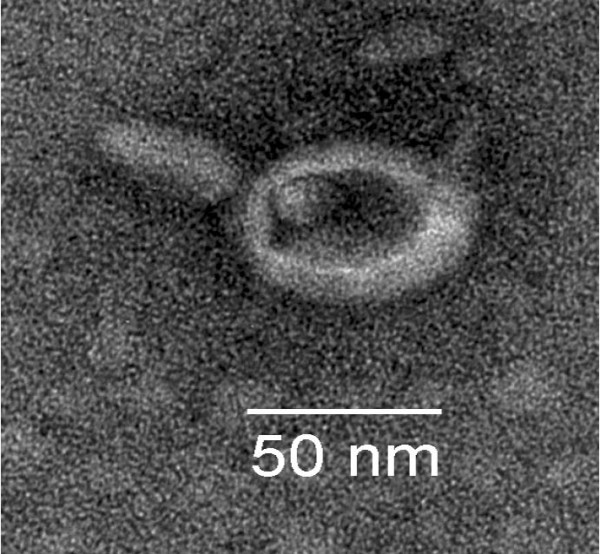
**Transmission electron micrograph of gonococcal bacteriophage**. Culture supernatants were precipitated with PEG 8000, dialyzed against TE buffer, added to a gold-grid, stained with Uranyl acetate and visualized on a Zeiss EM10CA electron microscope (160,000 magnification).

After identifying conditions where we could isolate supernatants containing phage DNA, we analyzed the culture supernatants for bacteriophage particles by electron microscopy. Phage particles (both filamentous and lambda-like) were readily visualized by electron microscopy. The data presented in figure 9 is an example of one of the lambda-like phage particles. This phage clearly contains a head and tail structure. Culture supernatants also contained numerous tail-less heads, and filamentous particles of varying lengths (data not shown). Given the diversity of phage encoding sequences in the genome of the producing strain, we did not attempt to determine which phage DNAs sequence was responsible for the production of the phage shown.

## Discussion

The existence of prophage DNA sequences in the bacterial chromosomes is very common [3]. It is also very common that several different prophage sequences can be present in the genome of a particular bacterial strain. For example, in eleven *Salmonella enterica *serovars Typhi and Typhimurium, two *Yersinia pestis *strains, *Shigella flexnerii*, two *Xylella fastidiosa*, and four *E. coli *strains that have been sequenced, each carries between 7 to 20 prophages (see review by Casjens [3]). Cryptic prophage DNA sequences have been found in several *N. meningitidis *strains as well [15,16]. Our analysis of nine DNA sequences of *N. gonorrhoeae *allowed us to identify the presence of two different types of bacteriophage DNA sequences in this species. The first group is represented by sequences that are homologous to the filamentous ssDNA prophages recently discovered in the chromosome of *N. meningitidis *[16,18]. The second group is represented by the sequences that show the high homology to the tailed dsDNA phages present in diverse groups of bacteria. The overall genetic organization of these phages resembles mostly that of P2 genome ld MACROBUTTON endnote+.cit [3]. This is in sharp contrast to *N. meningitidis *where dsDNA prophage sequences show homology to Mu-like phages representing a very specific group of phages [15].

NgoΦ1 and NgoΦ2 could encode functional bacteriophages since they seem to encode all of genes necessary for lytic growth. The ability to produce functional bacteriophage is further supported by the fact that we were able to detect the presence of some of these genes outside of the cells in a DNase resistant form (phage particles). We were able to visualize phage particles. We showed that some of the genes are biologically active. Since we were unable to further propagate phages on any of the strains tested, it suggests that each strain is expressing the appropriate lysogenic control genes.

It is much less probable that the phage belong to gene transfer agents that are tailed phage-like particles that encapsidate random fragments of the bacterial genome [3] since we were unable to detect the chromosomal sequences in the DNase resistant forms. Three of the dsDNA prophages present *in N. gonorrhoeae *FA1090 are probably defective forms of NgoΦ1 and NgoΦ2, being in the stage of complex decay of prophages. We believe that NgoΦ5 can be classified as a bacteriocin since it seems to encode mainly the genes of phage tail.

The presence of two types of biologically active prophage in the *N. gonorrhoeae *may form the molecular explanation for the observation of the formation of autoplaquing [12]. While Campbell and coworkers were clearly able to induce gonococci to produce products capable of inhibiting their growth, they were unable to demonstrate the propagation of phage. On the other hand, it is possible that autoplaquing does not result from phage propagation, but rather an alteration in an autoylytic mechanism within the cell, as suggested for autoplaquing in Myxococcus [26]. It could also be the result of down-regulation of the phage repressors and up-regulation of the phage encoded holin-endolysin lytic system that seem to be encoded by NgoΦ1 and NgoΦ2 prophages, in response to an environmental change.

To test for the presence of prophage sequences in different gonococcal strains we determined the presence of three different genes present in NgoΦ1 and NgoΦ2 using a PCR method, with the specificity of the PCR product verified by DNA sequence analysis. The results indicate that the prophage sequences detected in strain FA1090 are not present in all *N. gonorrhoeae *strains. Although, the specific PCR products encoding the large terminase subunits of NgoΦ1 and NgoΦ2 were formed using the chromosomal DNA from FA1090, MS11,1291 and WR302, the DNA encoding the holin gene (NGO0488) was not detected in 1291 and WR302. Similarly, we were unable to detect the presence of the DNA sequences representing phages NgoΦ3-NgoΦΦ5 in strains as WR220 or 1291. However, the lack of corresponding PCR products or very low amount of product formation, as in case of strain WR220 and 1291 the DNA encoding holin gene could be due to the differences in the DNA sequence encoding the genes in particular strains, thus changing the formation of the hybrids between primers and template DNA used for PCR reaction and the chromosomal DNA.

Presence of the prophage sequences in the bacterial genomes may have a profound effect on the pathogenicity of the host cell (see review by Wagner & Waldor, [27]) as well as on the population fitness [23]. Over time, a number of toxin genes have been found to be phage-encoded and it has become clear that toxin genes are only a part of diverse group of virulence factors encoded by bacteriophages [27]. We do not know whether any of the identified prophage genes of *N. gonorrhoeae *can be recognized as encoding bacterial toxins. On the other hand the properties of the NgoΦ1 and NgoΦ2 genes encoding putative repressors could have a profound effect on the fitness of *N. gonorrhoeae *strains. In *E. coli *the expression of the λ repressor inhibits the growth of cells in energy-poor environments, probably as an adaptive response to a host predation system [23]. We have shown that when *E. coli *is grown on a rich LB medium, the bacteria that express the repressor grows poorly and that induction of these genes inhibits of growth of cells, with high level of induction leading to cell death. This effect can be manifested by acting through the influence on the expression of different genes. The repressor of NgoΦ1 seems to inhibit the expression of the λ phage since even very low level of expression blocks the development of λ particles. These results suggest the tight control of genes encoding these repressors that would allow only very low levels of these proteins. The need for tight control of prophage gene expression is also evident from the activity of the NGO0488 encoding the holin. The activity of the gene has to be tightly controlled otherwise it is lethal for the cells and will results in the cell death.

Among different types of bacterial toxins that are phage encoded are the R- and F-type pyocins produced by *P. aeruginosa*. Pyocins are derived from a common ancestral origin with P2 phage and the λ phage respectively [22]. The gene organization of the R2 and F2 pyocins suggest that they are phage tails that have evolutionarily specialized to become bacteriocins. While the data presented in this paper do not show directly that these prophage sequences act as this type of toxin, the genes of NgoΦ4 encoding mainly the phage tail structural proteins could play such role.

## Conclusion

To our knowledge, this is the first demonstration of bacteriophage production by the gonococcus. Their role in pathogenicity is not known and has to be understood. The activity of the repressors on the chromosomal gene activity implies strict control of the level of their production. The ability to manipulate this level could be a potential method by which bacteria regulate their growth in the human body.

## Methods

### Bacterial strains, plasmids, phages and growth conditions

*Escherichia coli *Top 10: F^- ^*mcrA *Δ(*mrr-hsdRMS-mcrBC*) φ80 Δ*lac *ΔM15 Δ*lac*X74 *deoR recA1 araD139 *Δ(*araA*-*leu*) 7697 galU *galK λ*^s^-*rpsL endA1 nupG*, *E. coli *strain MM294 λ111 (*cI*857 Sam7), and *E. coli *3102 λ*cIts*857 were grown in Luria-Bertani broth (LB) at 37°C or 30°C [28]. Antibiotics included in media were used at the following final concentrations (μgml^-1^): ampicillin 100, kanamycin 10. *Neisseria *strains were grown in standardgonococcal medium (designated GCP if broth and GCK if agar) (Difco laboratories) plus Kellogg's growth supplements [29] and 0.042% sodium bicarbonate if in broth or in a 37°C CO_2 _incubator. *Haemophilus influenzae *strain Rd was grown in BHI (Difco) supplemented with 2 μg of NAD ml^-1 ^and 10 μg of hemin ml^-1 ^at 37°C [30]. Bacteriophage HP1 was originally obtained from R.D. Herriot. All HP1 phage manipulations were carried out as previously described [30] while those with λ phage as described by Sambrook *et al., *[28]. Plasmid pUC19 was purchased from MBI Fermentas. Plasmid pXYL20 was described previously [31]. Plasmid pMPMK6Ω was obtained from P. Mayer [24].

### Cloning of *N. gonorrhoeae *DNA fragments carrying the NgoΦ1 and NgoΦ2 prophage genes

A DNA fragment carrying ORF NGO0479 of *N. gonorrhoeae *FA1090 was amplified using primers RNGF1c and FNGF1CI. The resulting amplicon (717 bp) was cloned into pMPMK6Ω at the EcoRI and the PstI sites, resulting in the formation of plasmid pMPMK6cds*479*. A DNA fragment carrying ORF NGO1116 of *N. gonorrhoeae *FA1090 was amplified using primers F2Reg8L2 and F2Reg8Rt. The resulting amplicon (711 bp) was cloned into pMPMK6Ω and pMPMT4Ω at the EcoRI and PstI sites, resulting in the formation of plasmids pMPMT4cds1116 and pMPMK6cds1116 respectively. A DNA fragment carrying ORF NGO0488 was amplified using primers H0488T4F and HO488T4B. The resulting amplicon (150 bp) was cloned into pMPMK6Ω DNA into EcoRI and PstI sites, resulting in the formation of plasmid pMPMK6CDS488. All amplicons lacked their native promoters. Protein expression was from an inducible pBAD promoter.

### Cloning of the *H. influenzae *HP1 phage *p*L and *p*R promoters

The XylE cassette without any promoter sequence was derived from the plasmid pXYL20 and introduced into EcoRI and HindIII sites of pMPMK6Ω, resulting in the plasmid pMPMK6::XylE. The DNA fragments of phage HP1 carrying the pL and pR promoters [32] were amplified using HPPL-F and HPPL-R and HPPR-F and HPPR-R primers respectively. The resulting amplicons of 510 bp and 588 bp respectively were cleaved with EcoRI and BamHI and cloned into pMPMK6::XylE plasmid, resulting in the formation of plasmids pMPMK6::XylE::pL and pMPMK6::XylE::pR respectively. In both of these plasmids the pBAD promoter was removed and the XylE expression placed under control of the *p*L or *p*R promoters of HP1 phage.

### Detection of the prophage and phage sequences

For testing the presence of the prophage sequences in genomic DNA, PCR reactions were carried out using Pfu polymerase (MBI Fermentas) according to the manufacturer's instructions. The reaction mixture (25 μl) contained chromosomal DNA or phage DNA preparations. The reaction buffers contained: dATP, dGTP, dCTP and dTTP (0.2 mM each); forward and reverse primers (0.2 μM each); and 0.5 units of polymerase. Sequences of the primers used to amplify the chromosomal and prophage DNA and the PCR conditions are listed in Table 1. The specificity of the PCR products was confirmed by DNA sequencing the amplicons. All routine cloning procedures were carried out in accordance with protocols described in Sambrook *et al*. [28].

**Table 1:** List of primers used in this study

**Primer**	**Sequence (5'-3')**	**Description**	**PCR conditions**
F1TerD F	AATCACCATGCGCCAATCT GCAACGCGCTCGA	Amplifies the 5' end of NGO0495 (putative terminase) without any restriction sites	94°C, 5 min.; 2× (94°C, 48° 45 sec, 72°C 1 min); 30× (94°C, 45 sec.; 63°C,45 sec.; 72°C, 3 min.); 72°C, 10 min.
F1TerD R	TCCGTGTCCCATGTGCCG TCATCCAACACCAT	Amplifies the 3' end of NGO0495 (putative terminase) without any restriction sites	
F2Reg8L2	GC**GAATTC**GATGTCTGAA TTTAAAGACCGCCTGAAAG AG	Amplifies the 5' end of NGO1116 (putative repressor cI) with **EcoRI **site	94°C, 5 min.; 2×(94°C, 30 sec.; 48°C, 45 sec.; 72°C, 2 min.); 28× (94°C, 15 sec.; 50°C, 45 sec.; 72°C, 2 min.); 72°C, 10 min.
F2Reg8Rt	ATT**CTGCAG**TCACATCAAT CCAACACGCTCCACCAAA AG	Amplifies the 3' end of NGO1116 (putative repressor cI) with **PstI **site	
F2TerD F	AATATTCGTCGTGCAGCG GCCCCATTGCCACG	Amplifies the 5' end of NGO1098 (putative terminase) without any restriction sites	94°C, 5 min; 30× (94°C, 45 sec.; 63°C, 45 sec.); 72°C, 3 min.; 72°C, 10 min.
F2TerD R	CGGGCTGTTCAAGCCTTG CCGGTACAAGGTTA	Amplifies the 3' end of NGO1098 (putative terminase) without any restriction sites	
F2antrep F	GCCG**GAATTC**TCCAAGTT TTAAACTTTCAAC	Amplifies the 5' end of NGO1085 (putative antirepressor) with **EcoRI **site	94°C, 5 min.; 3×(94°C, 1 min.; 44°C, 45 sec.; 72°C, 3 min.); 27× (94°C, 1 min.; 59°C, 45 sec.; 72°C,3 min.); 72°C, 10 min
F2antrep R	TTAT**CTGCAG**TTACCTTAC CGTAGCCTTGCC	Amplifies the 3' end of NGO1085 (putative antirepressor) with **PstI **site	
F3dnaC F	ACGCGCTGGAAAAACGCA TC	Amplifies the 5' end of NGO1636 (putative replication protein DnaC) without any restriction sites	94°C, 2 min; 3× (94°C, 1 min.; 48°C, 45 sec.; 72°C, 3 min.28× (94°C, 45 sec.; 52°C, 45 sec.; 72°C, 2 min.); 72°C, 10 min.
F3dnaC R	TCCCAGTCGAACGGAATC AG	Amplifies the 3' end of NGO1636 (putative replication protein DnaC) without any restriction sites	
Fag4cI F	TCACTTTACCAAGCGCAGT TT	Amplifies the 5' end of NGO1013 (putative repressor cI) without any restriction sites	94°C, 2 min; 3× (94°C, 1 min.; 48°C, 45 sec.; 72°C, 3 min), 28×(94°C, 45 sec.; 50°C, 45 sec.; 72°C, 2 min.); 72°C, 10 min.
Fag4cI R	AATTAGGCTTGAACCAAGC AG	Amplifies the 3' end of NGO1013 (putative repressor cI) without any restriction sites	
Fag5cI F	TCTATCCAATCGAGGAACT GCC	Amplifies the 5' end of NGO0729 (putative repressor cI) without any restriction sites	94°C, 2 min; 28× (94°C, 45 sec.; 52°C, 45 sec.; 72°C, 2 min.); 72°C, 10 min.
Fag5cI R	CTACACGCCTTACAACCTT TCG	Amplifies the 3' end of NGO0729 (putative repressor cI) without any restriction sites	
RNFGF1c	TATCTGCAGTCTAGAACTG GCTCAAACGCCAT	Amplifies the 5' end of NGO0477 (putative repressor cI)	94°C, 2 min; 28× (94°C, 45 sec.; 52°C, 45 sec.; 72°C, 2 min.); 72°C, 10 min.
FNGF1C1	GCGAATTCGATGCCTTTTA TTATCGATTACCAATGC	Amplifies the 3' end of NGO0477	
HPPRL_F	CGCGGAATTCGACCCAGT CAAATATATTCC	Amplifies the 5' region of P_L _promoter of phage HP1	94°C, 5 min; 27×(94°C, 45 sec.; 56°C, 45 sec.; 72°C, 1 min.); 72°C, 10 min.
HPPRL_R	CGGGATCCTCGCAAAAAT CGCCGAAAG	Amplifies the 3' region of P_L _promoter of phage HP1	
HPPR_F	CGGGATCCCAGGTAACAC AGTGATATAAC	Amplifies the 5' region of P_R _promoter of Φ HP1	94°C, 5 min; 27× (94°C, 45 sec.; 56°C, 45 sec.; 72°C, 1 min.); 72°C, 10 min.
HPPR_R	CGCGGAATTCCCCGTATT GGTAAATAATGG	Amplifies the 3' region of P_R _promoter of Φ HP1	
HO488T4 F	GCGAATTCGATGGATACC CTGTTAAGCATCATCA	Amplifies the 5' region of putative holin of NgoΦ1	94°C, 2 min; 2× (94°C, 48° 45 sek, 72°C 1 min); 28 × (94°C, 45 sec.; 56°C, 45 sec.; 72°C, 1 min.); 72°C, 10 min.
HO488T4 B	ATTCTGCAGCTTAATATCC GAAGCCGTCGAAGT	Amplifies the 3' region of putative holin of NgoΦ1	

### Enzymes and chemicals

Restriction enzymes were purchased from MBI Fermentas and New England Biolabs. T4 DNA ligase, Pfu DNA polymerase and DNA and protein size markers were purchased from MBI Fermentas. Kits for the DNA purification and plasmid DNA isolation were purchased from A&A Biotechnology (Gdansk, Poland). All the chemicals used were reagent grade or better and were obtained from Sigma (St. Louis, MO), unless otherwise noted.

### XylE assay

Quantitative assays were performed as described by Braun & Stein, [31]. Briefly, 20 ml of LB was inoculated with an *E. coli *Top10 strain carrying the appropriate plasmid and grown at 37°C until a culture density of about 1 × 10^8 ^was achieved. The induction of the NGO1116 gene was achieved by the addition of arabinose to a final concentration of 0.1%, with additional incubation for 60 min at 37°C. Cells were harvested and resuspended in 2.5 ml of 50 mM potassium phosphate (pH 7.5), 20 mM EDTA-10% acetone (vol/vol) (pH 7.2) and 0.01% of Triton X-100. After 5 min of incubation on ice, the resulting crude lysate was clarified of cell debris by centrifugation at 4,000 × g for 5 min and then in a microfuge at 10,000 × g for 10 min. Assays were performed by diluting cell extracts in assay buffer (100 mM potassium phosphate, 0.2 mM catechol). Dilutions were chosen such that a linear change in absorbance at 375 nm was seen over time. XylE activity was calculated by linear regression of the slope over six time points. One microunit of XylE activity corresponds to the formation of 1 mM of 2-hydroxymuconic semialdehyde per min at 22°C. XylE activity was normalized against total protein concentration, as determined by the method of Bradford *et al. *[33], with bovine serum albumin (MBI Fermentas) as the standard.

### Phage techniques

The secreted form of phage and its DNA was prepared by standard phage preparation techniques [28]. Bacteria were collected by centrifugation from 200 ml of exponentially growing culture in GC medium, and DNA that was extracted from the cells was dissolved in 200 μl of TE buffer (10 mM Tris-HCl, 1 mM EDTA-Na pH, 8.0). After filtration through a 0.45 μM filter, the supernatant was treated for 3 h at 20°C with DNase I and RNaseA, (25 μg ml^-1^/ml each). Particles were precipitated by the addition of NaCl to a final concentration of 1 M and polyethylene glycol 8000 to 10%, incubation at 4°C overnight, and centrifugation at 12,000 g for 30 min. DNA from the presumptive phage particles was extracted with phenol, and the precipitated material was redissolved in 200 μl of TE buffer.

### *In silico *analysis

DNA and protein sequences were compared with the GenBank and SWISS-PROT databases on the BLAST server hosted by the National Center for Biotechnology Information [34]. The *N. gonorrhoeae *strain FA1090 genomic sequence was obtained from the University of Oklahoma's Advanced Center for Genome Technology [35] and the *N. meningitidis *strain Z2491 (serogroup A) genomic sequence (and the genomic sequence of the *N. meningitidis *strain FAM 18 from the Sanger Institute [36]. Other comparisons were performed using the BLAST tools at the NCBI web site [34]. Codon usage and codon frequency comparisons were performed using the CUSP and CODCMP programs at [37]. The G+C content of various chromosomal fragments was determined using the program COMPOSITION at [38]. ORFs were identified using GeneMark.hmm for PROKARYITIC (Version2.4) [39], EMBOSS [37], Glimmer [40], EasyGene 1.0 [41], ORF Finder[42], NCBI Conserved Domain Search [34], GeneImage Map for *Neisseria gonorrhoeae *[17], and EMBL-EBI (CpG content) [43]).

### Electron microscopy

An overnight culture of *N. gonorrhoeae *was diluted 30 times in fresh media and incubated with shaking for 2 hr. Mitomycin C was added (20 ng ml^-1 ^final concentration) and culture incubated in the dark for 3 hr. Chloroform was added and the culture shaken for 20 min. The cells and debris were removed by centrifugation for 20 min at 5000 rpm and the supernatant was filtrated through a 0.45 μm. Phage particles were precipitated by the addition of NaCl to a final concentration of 1 μM and polyethylene glycol 8000 to 10%, in the presence of CaCl_2 _to a final concentration of 1 mM and DNase I and RNaseA, (25 μg ml^-1 ^each) for 16 h at 4°C. The precipitate was centrifuged for 30 min at 12 500 rpm at 4°C. The pellet was resuspended in TE buffer. Phage were stained with uranyl acetate (2%) for 30 sec prior to visualization on a Zeiss EM10CA microscope (80 kv).

## Authors' contributions

AK participated in the molecular and phage biology experiments participated in the sequence alignment. MAP performed the sequence alignments. MM participated in the molecular and phage biology experiments. TKM performed the electron microscopy. AP helped conceive the study, participated in the design of the study, participated in the molecular and phage biology experiments, participated in the sequence alignment, and participated in the drafting of the manuscript participated drafted the manuscript. DCS helped conceive the study, participated in the design of the study and participated in the drafting of the manuscript. All authors have read and approve this manuscript.
